# Investigation of the inhibitory effects of the telomere-targeted compounds on glutathione S-transferase P1

**DOI:** 10.1007/s00210-025-03882-w

**Published:** 2025-02-15

**Authors:** Mehmet Ozcan, Ayse Burus, Ilgen Mender, Z. Gunnur Dikmen, Sergei M. Gryaznov, Turgut Bastug, Yasemin Bayazit

**Affiliations:** 1 Department of Medical Biochemistry, Faculty of Medicine, Zonguldak Bulent Ecevit University, Zonguldak, Turkey; 2 Department of Medical Biochemistry, Faculty of Medicine, Hacettepe University, Ankara, Turkey; 3 MAIA Biotechnology, Inc., Chicago, IL 60606 USA; 4 Department of Biophysics, Faculty of Medicine, Hacettepe University, Ankara, Turkey

**Keywords:** Glutathione S-transferase P1, Telomere-targeted drugs, 6-thio-2′-deoxyguanosin, Drug resistance

## Abstract

Glutathione S-transferase P1 (GSTP1) plays a significant role in cancer progression and chemotherapy resistance, with its overexpression diminishing chemotherapeutic efficacy across various tumor types. This study evaluates the inhibitory effects of 6-thio-2′-deoxyguanosine (6-thio-dG) and its dimeric form (6-thio-2′-dG-Dimer) on GSTP1. Enzyme inhibition assays with recombinant human GSTP1, kinetic analysis, molecular docking, and molecular dynamic simulations were employed. Enzymatic assays were performed in 0.1 M phosphate buffer (pH 6.5) at 30 °C, containing 1 mM EDTA, 1 mM GSH, and 1 mM CDNB. The compounds 6-thio-dG and its dimer were dissolved in 2.5% DMSO for the experiments. The IC₅₀ values indicated that the dimer exhibited a higher potency (IC₅₀: 0.339 μM) than the monomer (IC₅₀: 15.14 μM). Kinetic analysis revealed noncompetitive inhibition with glutathione (*Ki*: 12.26 μM) and mixed inhibition with CDNB (*Ki*: 11.41 μM) for the monomer, whereas the dimer showed mixed inhibition with glutathione (*Ki*: 0.972 μM) and competitive inhibition with CDNB (*Ki*: 0.723 μM). Molecular docking confirmed the higher binding affinity of the dimer (binding energy: − 7.9 kcal/mol, *Ki*: 1.595 μM) compared to the monomer (binding energy: − 6.2 kcal/mol, *Ki*: 28.21 μM). The dimer form of 6-thio-dG shows strong potential to enhance chemotherapeutic efficacy by effectively inhibiting GSTP1 and overcoming drug resistance. Its superior inhibitory properties make it a valuable candidate for targeted cancer therapies.

**Graphical abstract:**

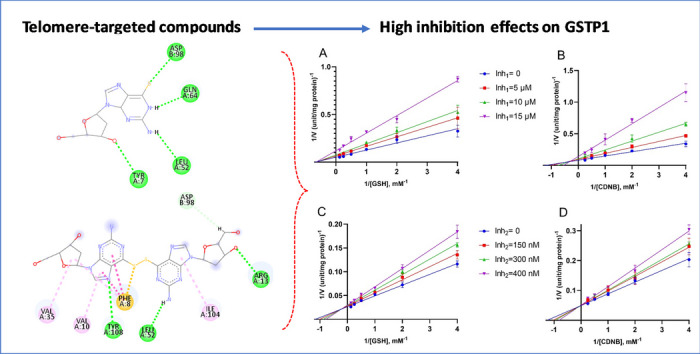

## Introduction

Glutathione S-transferase P1 (GSTP1) is a key enzyme in the cellular defense system, primarily involved in the conjugation of glutathione to various substrates, taking role in the detoxification of reactive electrophiles and oxidative stress products. GSTP1 is often overexpressed in cancers such as lung, breast, and prostate, where it has been linked to chemotherapy resistance (Chatterjee and Gupta 2018; Cui et al. 2020). The enzyme’s overexpression in cancer cells enhances their resistance to chemotherapeutic agents by neutralizing reactive oxygen species (ROS) and promoting drug detoxification, thereby diminishing their cytotoxic effects (Gu et al. 2024). The role of GSTP1 in promoting drug resistance and tumor survival makes it a promising therapeutic target for cancer treatment (Townsend and Tew 2003; Singh and Reindl 2021).

Because of GSTP1’s role in drug resistance and tumor survival, targeting this enzyme has become a promising therapeutic approach (Ozcan et al. 2024). Inhibiting GSTP1 activity has the potential to amplify chemotherapy’s cytotoxic effects by reducing drug detoxification and increasing oxidative stress in tumor cells (Ozcan et al. 2019, 2021). For instance, the small molecule inhibitor TLK199 has shown promise in sensitizing cancer cells to chemotherapy by selectively targeting GSTP1, improving the efficacy of drugs like cisplatin in ovarian cancer models (Lv et al. 2023). Similarly, ezatiostat has been explored for its GSTP1 inhibitory activity, enhancing chemotherapy sensitivity in acute myeloid leukemia cells (Raza et al. 2012). Ethacrynic acid, a known GSTP1 inhibitor, binds to the enzyme’s active site, thereby reducing drug detoxification. This leads to increased drug accumulation in cancer cells, which can enhance the effectiveness of chemotherapy agents like doxorubicin and cyclophosphamide (Tew and Gaté, 2001; Potęga 2022). While several other small-molecule inhibitors have been developed to target GSTP1, challenges remain in finding potent and selective compounds capable of effectively inhibiting this enzyme in cancer cells.

In addition to GSTP1 inhibition, targeting telomerase presents another approach to limit cancer cell proliferation. Telomerase maintains telomere length, allowing many cancer cells to bypass normal aging and divide indefinitely (Mender et al. 2015b). 6-thio-2′-deoxyguanosine (6-thio-dG), a telomere-targeted compound with anticancer potential, is incorporated into the telomeric end by telomerase, resulting in telomere dysfunction (Mender et al. 2015a, b). This process inhibits tumor growth and reduces metastatic burden in preclinical mouse models (Mender et al. 2015b; Sengupta et al. 2018). With the pivotal role of telomeres in cancer cell survival, telomere-targeted agents like 6-thio-dG and its derivatives are emerging as valuable options in cancer therapy.

This study focuses on the potential of 6-thio-2′-deoxyguanosine (6-thio-dG) and its dimer form to inhibit GSTP1, leveraging both experimental and computational techniques. Molecular docking simulations were conducted to analyze the binding interactions, affinities, and inhibition constants of these compounds with GSTP1. The results showed that 6-thio-dG and its dimer form exhibited strong inhibitory effects, with a high degree of alignment between experimental data and computational predictions. These findings underscore the potential of 6-thio-dG and its dimer form as GSTP1 inhibitors, highlighting their promise in enhancing cancer treatment and overcoming drug resistance.

## Materials and method

### Materials

The telomere-targeted compounds, 6-thio-2′-deoxyguanosine (6-thio-dG) and its dimer form (6-thio-2′-dG-Dimer), were obtained from MAIA Biotechnology (Chicago, USA). Unless otherwise specified in this article, all chemicals and reagents were purchased from Millipore Sigma (St. Louis, MO, USA).

### Human GST P1 expression and purification

The human GST P1-1 gene from a K562 erythroleukemia cDNA library (GSTP1*A allele) was cloned into the pKXHP1 plasmid. This plasmid was transformed into *E. coli* XL-1 Blue cells via heat shock, grown on agar plates, and a single colony was cultured overnight at 37 °C in 2YT medium. A fresh culture was prepared with a 1:1000 dilution and induced with 0.2 mM IPTG at an OD600 of ~ 0.4, followed by 16 h of incubation. After harvesting by centrifugation, cells were lysed in a buffer containing Tris-HCl, EDTA, DTT, and lysozyme, then sonicated and centrifuged. The resulting supernatant was applied to a Nickel-Sepharose column, washed, and eluted with 300 mM imidazole in a washing buffer. The eluate was dialyzed in Tris-HCl buffer with DTT and EDTA for final purification.

### Inhibition studies

The enzyme activity of GSTP1 was measured using a spectrophotometric assay based on the conjugation of glutathione (GSH) to 1-chloro-2,4-dinitrobenzene (CDNB). The inhibitory effects of 6-thio-dG and its dimer were evaluated by adding these compounds at varying concentrations and analyzing their impact on GSTP1 activity. All reactions were conducted at pH 6.5 in 100 mM phosphate buffer at 30 °C to ensure optimal enzymatic activity. The progress of the reaction was monitored by measuring the absorbance change at 340 nm, indicating the formation of the GSH-CDNB conjugate. Absorbance readings were recorded over 5 min using a SpectraMax M2 microplate reader. Percentage inhibition at each concentration was calculated, and IC₅₀ values were determined using GraphPad Prism 8.4. The IC₅₀ calculation in this study was performed using the formula implemented in GraphPad Prism, which utilizes the four-parameter logistic (4PL) model. The formula used is: Fifty = (Top + Baseline)/2 and Y = Bottom + (Top–Bottom)/(1 + 10^((LogAbsoluteIC50—X) * HillSlope + log((Top–Bottom)/(Fifty—Bottom)—1)))). In this model, the Top represents the maximal response, the Baseline is the minimum response, the Bottom is the lowest value of the dose-response curve, LogAbsoluteIC50 is the logarithmic value of the IC₅₀, X is the independent variable (concentration), and HillSlope indicates the steepness of the curve.

### Determination of kinetic inhibition parameters

To assess the inhibition type and calculate the inhibition constant (Ki) values for the compounds, kinetic inhibition studies were performed with GSTP1. In each assay, one substrate (either GSH or CDNB) was maintained at a near-saturating concentration to prevent it from limiting the reaction rate, while the concentration of the other substrate was varied across a range, alongside different concentrations of the inhibitor (6-thio-dG or its dimer).

By varying the concentration of one substrate while keeping the other at near-saturation, we assessed how each inhibitor interacted with GSTP1 under various substrate conditions. Lineweaver-Burk plots were used to analyze these interactions, allowing for the determination of the inhibition type (e.g., competitive, non-competitive, or mixed) and the Ki values for each compound. These kinetic parameters were calculated using GraphPad Prism, providing a comprehensive understanding of how the inhibitors influence GSTP1 enzymatic activity.

### Docking analysis

The molecular structures of 6-thio-dG and its dimer were drawn and energy-minimized using the Avogadro program to ensure accurate docking parameters for the inhibitors (Hanwell et al. 2012). The crystal structure of Glutathione S-transferase P1 (GSTP1) was retrieved from the Protein Data Bank (PDB) under the code 2GSS (resolution: 1.9 Å, R-factor: 0.209, R-free: 0.229) (Oakley et al. 1997). Before docking, water molecules and non-protein structures were removed, and hydrogen atoms were added to the protein structure, with Gasteiger charges assigned for accurate docking. The active site was identified based on the binding site of ethacrynic acid, a known GSTP1 inhibitor. The coordinates of the active site were x: 9.2316, y: 3.21369, and z: 28.8571, defined within a cubic region of 20 Å × 20 Å × 20 Å. Docking was performed using AutoDock Vina (version 1.2.5) (Trott and Olson 2010; Eberhardt et al. 2021), and binding affinities were calculated using the Lamarckian Genetic Algorithm.

### Docking process validation

Validation of the docking procedure is a crucial step in ensuring the accuracy of molecular docking results. Various validation approaches have been described in the literature (Matore et al. 2022, 2023). In this study, the pose selection method was employed for docking validation using the reference crystal structure of GSTP1 in a complex with ethacrynic acid. The co-crystallized ligand was re-docked into the active site, and the root-mean-square deviation (RMSD) between the original and re-docked ligand poses was calculated. The RMSD value was found to be below the 2 Å threshold, confirming the reliability of the docking protocol.

### Determination of molecular interactions

Post-docking, molecular interactions between GSTP1 and 6-thio-dG or its dimer (used as inhibitors) were analyzed. The interactions, including hydrogen bonds and hydrophobic contacts, were visualized and examined with the Discovery Studio software. These analyses provided insights into the binding dynamics and interactions of GSTP1 with the inhibitors.

### Molecular dynamic simulations

The docking results were used as initial configurations for molecular dynamic (MD) simulations. The protein-ligand complex was placed in a water box with dimensions of 82 × 82 × 87 Å^3^, containing 17,200 water molecules. To achieve a physiological ion concentration of 150 mM and neutralize the system, 54 Na^+^ and 48 Cl^−^ ions were added. MD simulations were performed using NAMD 2.7 software (Phillips et al. 2020), with the CHARMM biomolecular force field and the TIP3P water model (Huang and MacKerell Jr 2013). The system structure was generated using the PSFgen utility from the VMD program (Humphrey et al. 1996).

Once the system was constructed, it underwent a two-stage equilibration process. In the first stage, the coordinates of the protein and ligand atoms were fixed, and the system was equilibrated at 1 atm pressure to achieve the correct water densities. The *x* and *y* dimensions of the simulation box were constrained to 80 Å, while pressure coupling was applied in the *z*-direction (with an average *z* length of 86 Å). In the second stage, the protein and ligand were gradually relaxed over a 4-ns period by progressively reducing restraints on their atoms in multiple steps. After the restraints were removed, both the protein and ligand were treated as fully flexible.

During the MD simulations, the temperature was maintained at 300 K using the Langevin damping method, and the pressure was kept at 1 atm using the Langevin piston method. Lennard-Jones interactions were disabled for distances greater than 12 Å, with a switching distance of 10 Å. Electrostatic interactions were calculated without truncation using periodic boundary conditions and the particle-mesh Ewald method. A 2 fs time step was employed for all simulations, which were carried out for a total of 50 ns.

## Results

### Evaluation of IC₅₀ values

The inhibitory effects of 6-thio-2′-deoxyguanosine (6-thio-dG) and its dimer form on GSTP1 were evaluated by determining the IC₅₀ values. As shown in Table 1, 6-thio-dG exhibited an IC₅₀ of 15.14 μM, while the dimer form demonstrated a much lower IC₅₀ value of 0.339 μM. This suggests that the dimer form has a significantly higher potency in inhibiting GSTP1 compared to the monomer.

**Table 1:** IC₅₀ values of 6-thio-2′-deoxyguanosine (6-thio-dG) and its dimer form (6-thio-2′-dG-Dimer) against GSTP1

Compound	Molecular Structure	IC_50_
6-thio-2′-deoxyguanosine (6-thio-dG)		15.14 μM
6-thio-2′-dG-Dimer		0.339 μM

### Enzyme kinetics and inhibition type analysis

**Fig. 1:**
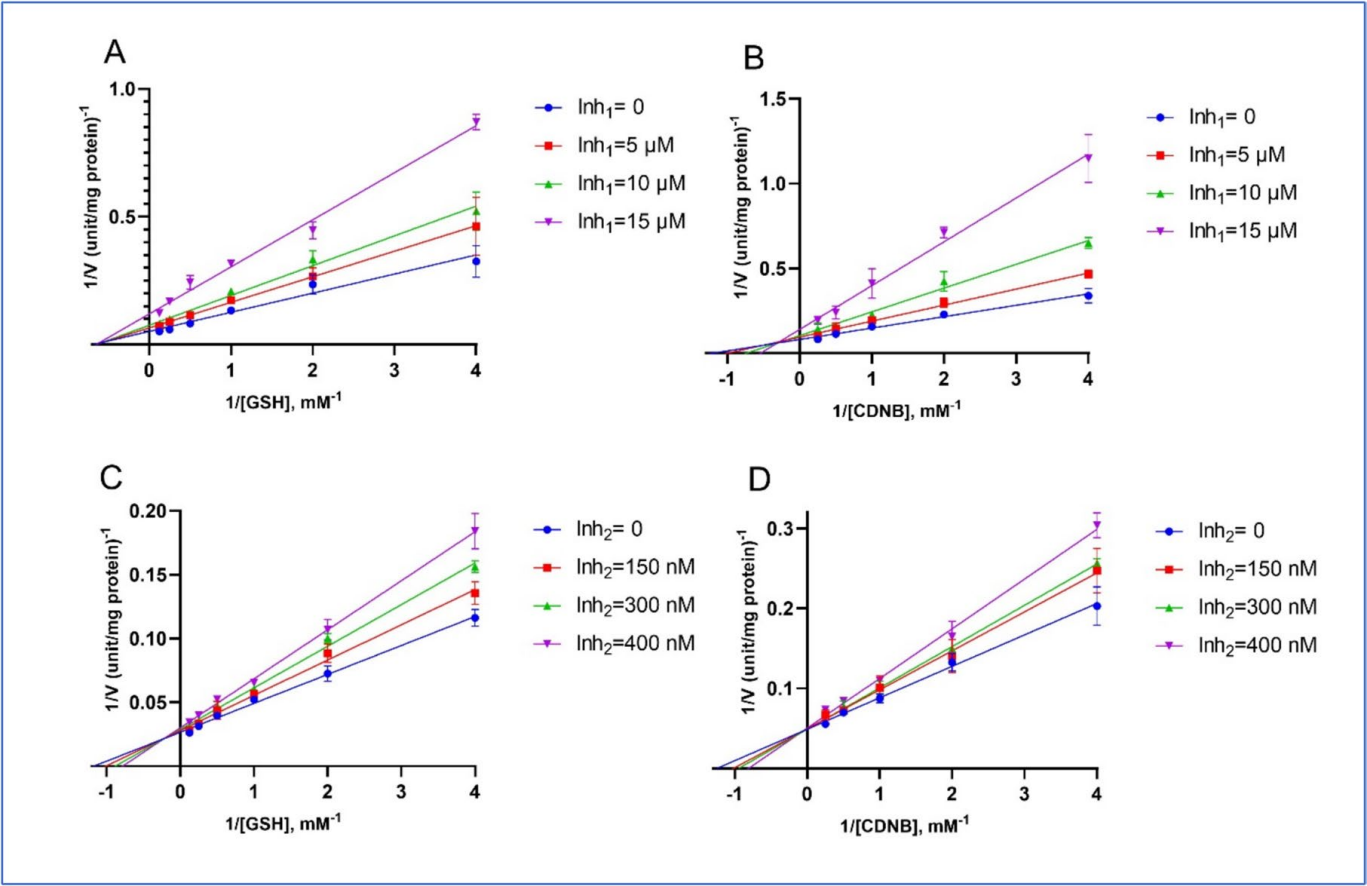
Lineweaver-Burk plots illustrating GSTP1 inhibition with varied GSH (**A**, **C**) and CDNB (**B**, **D**) concentrations. Panels **A** and **B** depict inhibition by Inh₁ (6-thio-dG) at 0, 5, 10, and 15 μM. Panels **C** and **D** show inhibition by Inh₂ (6-thio-2′-dG-Dimer) at 0, 150, 300, and 400 nM. Error bars represent standard deviations (*n* = 3)

The inhibitory effects of 6-thio-dG and its dimer form (6-thio-2′-dG-Dimer) on GSTP1 were further examined using Lineweaver-Burk plots, shown in Fig. 1. These plots were generated by varying the concentrations of substrates GSH and CDNB with different inhibitor concentrations. For 6-thio-dG (Inh₁), panels A and B in Fig. 1 show inhibition at concentrations of 0, 5, 10, and 15 μM, while panels C and D depict the dimer form (Inh₂) at concentrations of 0, 150, 300, and 400 nM. The corresponding *K*_*i*_ values and inhibition types are summarized in Table 2. The results show that 6-thio-dG had a *K*_*i*_ of 12.26 μM with GSH and 11.41 μM with CDNB, displaying non-competitive inhibition with GSH and mixed-type inhibition with CDNB. The dimer form showed a K_i_ of 0.972 μM with GSH and 0.723 μM with CDNB, indicating mixed-type inhibition with GSH and competitive inhibition with CDNB. These findings suggest that dimerization increases the specificity and binding efficiency of the inhibitor.

**Table 2:** Inhibition constants (*Ki*) and inhibition types of compounds for GSTP1-1 concerning GSH and CDNB as varied substrates

Compound	*Ki* ^GSH^	*Ki* ^CDNB^	Type of inhibition	
			GSH	CDNB
6-thio-dG	12.26 μM	11.41 μM	Noncompetitive	Mixed
6-thio-2′-dG-Dimer	0.972 μM	0.723 μM	Mixed	Competitive

### Molecular docking and binding energy analysis

Molecular docking studies were conducted to assess the interactions between GSTP1 and the compounds, focusing on their binding energies and inhibition constants (*K*_*i*_), as shown in Table 3. The inhibition constant (Ki) was calculated from the binding energy (ΔG) using the equation Ki = e^ΔG/RT^ where R represents the universal gas constant (1.985 × 10^−3^ kcal mol^−1^ K^−1^) and T is the temperature set at 298.15 K. The dimer form exhibited the highest binding affinity, with a binding energy of − 7.9 kcal/mol and an inhibition constant of 1.595 μM, significantly higher than both the monomer form (binding energy of − 6.2 kcal/mol, K_i_ of 28.21 μM) and the reference inhibitor, ethacrynic acid (binding energy of − 6.6 kcal/mol, K_i_ of 14.35 μM). These results suggest that the dimer form could be a more effective GSTP1 inhibitor, possibly due to stronger binding interactions within the GSTP1 active site.

**Table 3:** Binding energy and calculated inhibition constant (*Ki*) for monomer and dimer forms of the compound and ethacrynic acid as a reference inhibitor

Compound	Binding energy (kcal/mol)	Calculated *Ki* (μM)
6-thio-dG	− 6.2	28.21
6-thio-2′-dG-Dimer	− 7.9	1.595
Ethacrynic acid	− 6.6	14.35

### Analysis of binding interactions

**Fig. 2:**
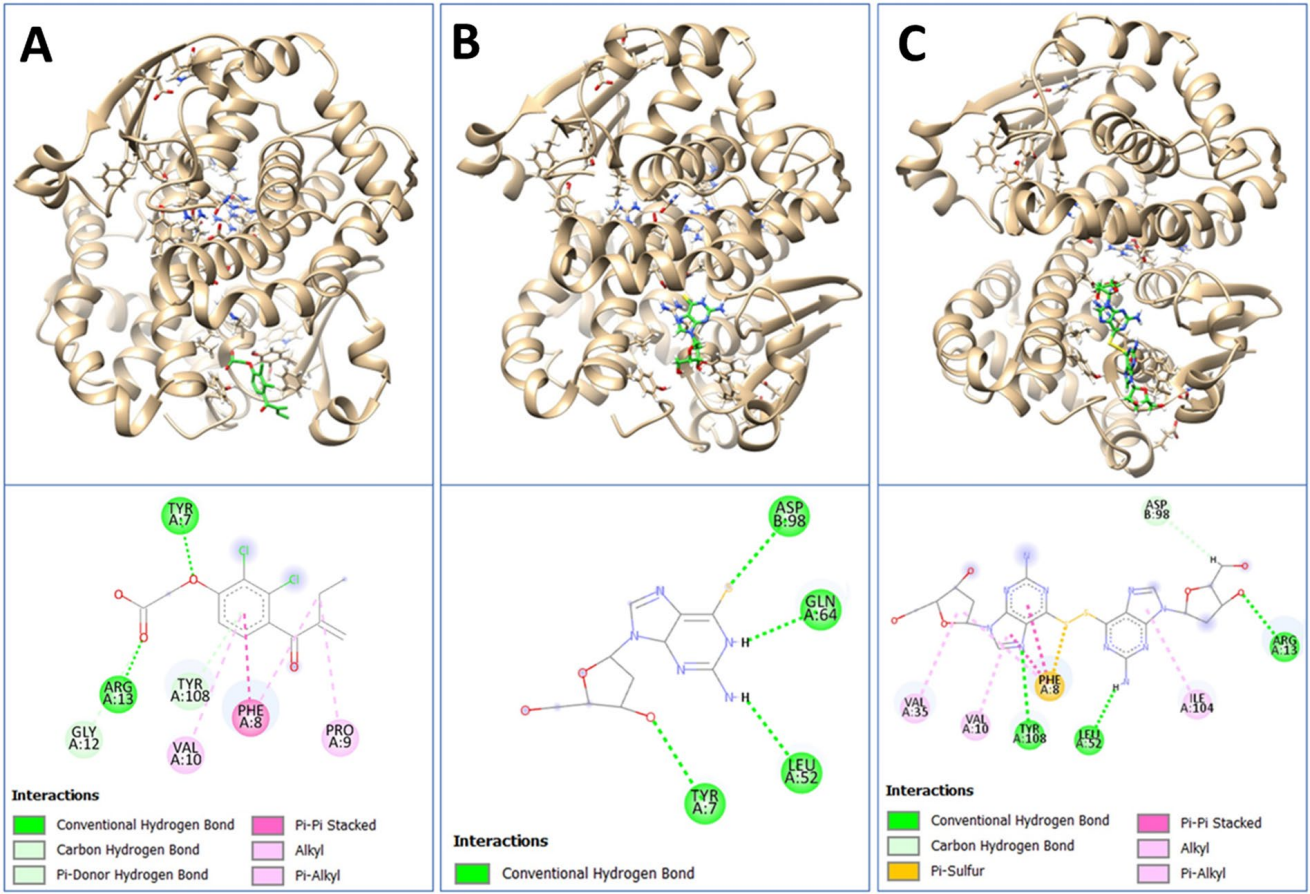
Molecular docking interactions of GSTP1 with ethacrynic acid, 6-thio-2′-deoxyguanosine, and its dimer form. **A** Ethacrynic acid, a known GSTP1 inhibitor, in the GSTP1 binding site, with its 3D spatial orientation (top) and 2D interaction map (bottom) displayed. **B** 6-Thio-2′-deoxyguanosine positioned in the GSTP1 active site. **C** Dimer form of 6-thio-2′-deoxyguanosine in the GSTP1 binding pocket. The 2D diagrams illustrate conventional hydrogen bonds, carbon hydrogen bonds, π-π stacked interactions, alkyl and π-alkyl interactions, color-coded per the legend

Figure 2 provides a detailed view of the binding interactions between GSTP1 and each inhibitor, highlighting the diversity and extent of interactions that contribute to the inhibitory potential of each compound.

Panel A presents the 3D structure and 2D interaction map of ethacrynic acid, a well-established GSTP1 inhibitor. The interaction profile reveals multiple hydrogen bonds and hydrophobic interactions with key residues such as ARG13, TYR7, TYR108, and PHE8. These interactions stabilize ethacrynic acid within the GSTP1 active site, aligning with its known mechanism of action in reducing GSTP1 activity by interfering with its catalytic function.

Panel B illustrates the binding of the monomeric form of 6-thio-2′-deoxyguanosine (6-thio-dG) within the GSTP1 active site. The interaction profile shows limited hydrogen bonding and van der Waals forces, primarily involving residues ASP98, GLN64, LEU52, and TYR7. Compared to ethacrynic acid, this compound exhibits a weaker interaction profile, which may correlate with a lower binding affinity and higher IC₅₀ value, suggesting reduced GSTP1 inhibition.

Panel C highlights the dimeric form of 6-thio-2′-deoxyguanosine (6-thio-2′-dG-Dimer), which demonstrates a more extensive network of interactions within the GSTP1 binding site. The 3D structure and 2D interaction map reveal numerous π-π stacking, π-alkyl, and hydrogen bond interactions with residues around the active site. Notably, the presence of π-anion and π-sulfur interactions suggests strong non-covalent stabilization, contributing to the dimer’s lower IC₅₀ and *Ki* values compared to the monomer.

Overall, the molecular interactions presented in Fig. 2 support the observed kinetic and inhibition data, reinforcing the conclusion that the dimer form of 6-thio-2′-deoxyguanosine (6-thio-2′-dG-Dimer) exhibits a stronger affinity for GSTP1, potentially enhancing its effectiveness as a therapeutic GSTP1 inhibitor.

### Evaluation of molecular dynamic simulations

**Fig. 3:**
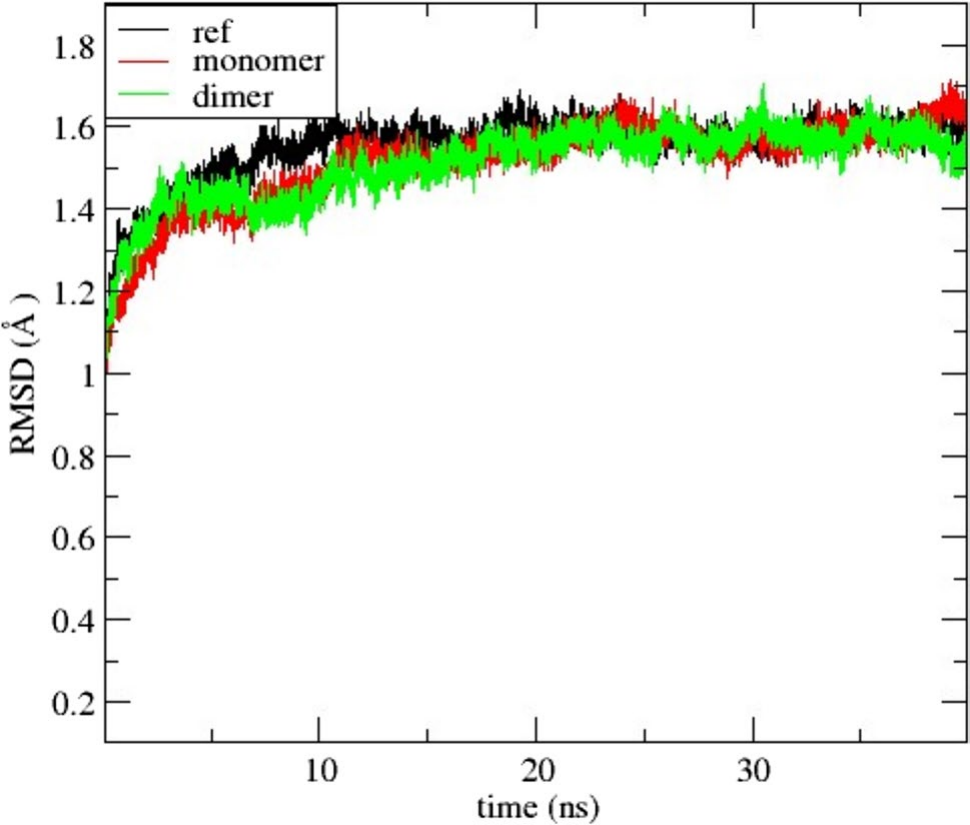
RMSD of protein atoms for reference (black), monomer (red), and dimer (green) systems

The Root Mean Square Deviation (RMSD) plot for the protein atoms over the last 20 ns of simulation for all three systems is shown in Fig. 3. The RMSD values for all systems stabilize around 1.5 Å, indicating that the systems have reached equilibration. After completing the MD simulations, MMPBS (Molecular Mechanics Poisson-Boltzmann Surface Area) calculations were performed to determine the free energies of the three systems (Liu and Hou 2016). These calculations were carried out from 30 to 50 ns. The results show that the monomer system exhibits the best binding affinity with a free energy of − 2.1 kcal/mol, followed by the dimer system, which has a free energy of − 0.6 kcal/mol. The reference system, in comparison, shows the least binding affinity with a free energy of − 0.3 kcal/mol.

## Discussion

This study investigates the inhibitory potential of telomere-targeted compounds, specifically 6-thio-dG and its dimer form, on GSTP1, a key enzyme implicated in cancer progression and chemoresistance. GSTP1 is frequently overexpressed in various cancers, where it serves as a protective mechanism for cancer cells by detoxifying reactive molecules and reducing oxidative stress (Chatterjee and Gupta 2018; Cui et al. 2020). This activity effectively shields cancer cells from the cytotoxic effects of chemotherapeutic agents. Given GSTP1’s prominent role in drug resistance, targeting this enzyme has emerged as a promising strategy in cancer therapy. By inhibiting GSTP1’s detoxifying function, chemotherapy drugs can exert greater cytotoxic effects, potentially enhancing therapeutic outcomes (Ozcan et al. 2024). Our findings align with and extend previous research on GSTP1 inhibition, such as studies involving ethacrynic acid (Tew and Gaté, 2001; Potęga 2022) and TLK199 (Lv et al. 2023), which demonstrated the ability of these inhibitors to increase cancer cell sensitivity to chemotherapy. However, unlike traditional inhibitors, telomere-targeted compounds like 6-thio-dG offer dual functionality by both disrupting GSTP1 activity and inducing telomere dysfunction (Mender et al. 2015a, b), which can further weaken cancer cell viability. This dual mechanism may enhance the therapeutic impact compared to previous GSTP1 inhibitors, suggesting a unique advantage for telomere-targeted approaches in overcoming drug resistance.

In this study, the novel dimeric form of 6-thio-dG has an impressive IC_50_ value of 339 nM, substantially lower than other reported GSTP1 inhibitors, highlighting its potent activity. For instance, LAS17, one of the most effective known inhibitors, has an IC_50_ of 500 nM and works by covalently binding to tyrosine residues on GSTP1 (Louie et al. 2016), showing significant efficacy in reducing tumor growth in xenograft models with minimal toxicity. Another covalent inhibitor, GS-ESF, targets the G-site of GSTP1(Shishido et al. 2017), while NBDHEX also acts covalently to modify the enzyme’s active site and trigger apoptosis in cancer cells (Tentori et al. 2011; De Luca et al. 2014). Ethacrynic acid (EA) is a widely used inhibitor of GSTP1, known for its potent inhibitory effects on this enzyme. It has been applied in various therapeutic contexts, particularly in cancer treatments, due to its ability to sensitize tumor cells to chemotherapy by inhibiting GSTP1, which is involved in drug resistance. Its IC_50_ value for GSTP1 inhibition varies within the micromolar range, depending on experimental conditions (Tew and Gaté, 2001; Musdal et al. 2013; Singh and Reindl 2021; Potęga 2022). Despite its efficacy, EA’s therapeutic use is limited by its diuretic side effects and lack of specificity, prompting the development of derivatives and analogs to improve its selectivity and potency (Tew and Gaté, 2001; Musdal et al. 2013). However, unlike these inhibitors, the dimeric form of 6-thio-dG operates through a competitive inhibition mechanism against CDNB (1-chloro-2,4-dinitrobenzene), a standard GST substrate. This competitive binding may offer advantages over covalent inhibition by enhancing specificity and reducing the risk of non-specific interactions, which can limit covalent inhibitors’ therapeutic use. Therefore, our compound’s significantly lower IC_50_, coupled with its competitive inhibition profile, suggests it could be a promising GSTP1 inhibitor with both higher potency and a potentially safer therapeutic profile.

Kinetic inhibition analysis further clarified the mechanisms through which these compounds interact with the enzyme. Our results revealed that 6-thio-dG exhibits non-competitive inhibition concerning the GSH substrate while showing mixed-type inhibition with CDNB. In contrast, the dimer form demonstrated mixed-type inhibition with GSH and competitive inhibition with CDNB. This differential inhibition profile suggests a flexible binding mechanism, particularly for the dimer, which may selectively modulate GSTP1 activity depending on substrate concentration. Such substrate-dependent inhibition profiles could offer distinct therapeutic advantages (Crettol et al. 2010; Lam 2019), as the dimer form may provide optimal inhibition across diverse cellular environments with varying levels of GSH and CDNB.

Our findings demonstrate that both 6-thio-dG and its dimer form (6-thio-2′-dG-Dimer) effectively inhibit GSTP1, with the dimer showing markedly greater potency. This conclusion is supported by our IC₅₀ and Ki data, where the dimer exhibits significantly lower values compared to the monomer. The potency of the dimerized form may be attributable to its greater molecular weight and structural complexity, which likely enhance binding affinity and stability within the GSTP1 active site. These findings align with existing literature that suggests multivalent inhibitors generally show increased potency due to enhanced interactions with target proteins, providing a strategic approach to the design of GSTP1 inhibitors [24–26].

In addition to the experimental results, molecular docking analysis offered crucial insights into the interaction dynamics between GSTP1 and these compounds. Our simulations revealed that the dimer form (6-thio-2′-dG-Dimer) had a more stable and extensive interaction network within the GSTP1 active site compared to the monomer, including π-anion, π-sigma, and hydrogen bonding interactions essential for enhanced binding affinity. This detailed interaction profile highlights the dimer’s superiority over the monomer, suggesting that dimerization could be a promising strategy for developing more potent GSTP1 inhibitors, especially for applications targeting chemoresistant cancers. Moreover, the binding values obtained from the docking analysis and the experimentally Ki values were in good agreement, further supporting the validity of our findings.

Despite these promising findings, this study has a few limitations. While molecular docking provides insights into binding interactions, it cannot fully replicate the complexity of living cell environments, making further in vitro and in vivo studies essential to confirm the efficacy of these compounds in cancer cell models and animal studies. Additionally, the effects of 6-thio-dG and its dimer on other cellular targets were not examined, leaving the potential for off-target interactions that could impact safety profiles. Moreover, only GSH and CDNB inhibition assays were employed; while these are commonly used in GSTP1 research, they may not capture the full range of GSTP1’s physiological substrates in human cells. Expanding to additional substrates would provide a more comprehensive view of the inhibition mechanisms of 6-thio-dG and its dimer. Furthermore, although this study highlighted the potential of these compounds to counter chemotherapy resistance, the combined effects of 6-thio-dG with standard chemotherapeutic agents remain unexplored, warranting future studies on synergistic interactions to establish optimal combination therapies.

## Conclusion

This study highlights 6-thio-dG and its dimer as promising GSTP1 inhibitors with the potential to counteract chemoresistance in cancer. Using biochemical assays, kinetic analysis, and molecular docking studies, we demonstrated that the dimer form exhibits superior binding affinity and inhibitory potency compared to both the monomer form and the reference inhibitor, ethacrynic acid. These findings support the continued development of telomere-targeted GSTP1 inhibitors for cancer therapy. It has been shown that the nucleoside analog 6-thio-dG leads to tumor regression through innate and adaptive immune-stimulatory responses and overcomes resistance to checkpoint inhibitors (PD-L1/PD-1blockade resistance) in mouse models. Hence, this is the first study showing the inhibitory effects of both 6-thio-dG 6-thio-2′-dG-Dimer on the GSTP1 enzyme. Future research should include comprehensive in vitro and in vivo studies to assess these compounds’ effects in a dynamic cellular environment and verify their selectivity for GSTP1 in cancerous versus normal cells. Together, these efforts could pave the way for novel, targeted strategies in cancer therapy, offering new hope in the fight against immunotherapy-resistant advanced cancers.

## Data Availability

All source data for this work (or generated in this study) are available upon reasonable request.
